# Finding biomedical categories in Medline^®^

**DOI:** 10.1186/2041-1480-3-S3-S3

**Published:** 2012-10-05

**Authors:** Lana Yeganova, Won Kim, Donald C Comeau, W John Wilbur

**Affiliations:** 1 National Library of Medicine, National Institutes of Health, Bethesda, MD 20894, USA

## Abstract

**Background:**

There are several humanly defined ontologies relevant to Medline. However, Medline is a fast growing collection of biomedical documents which creates difficulties in updating and expanding these humanly defined ontologies. Automatically identifying meaningful categories of entities in a large text corpus is useful for information extraction, construction of machine learning features, and development of semantic representations. In this paper we describe and compare two methods for automatically learning meaningful biomedical categories in Medline. The first approach is a simple statistical method that uses part-of-speech and frequency information to extract a list of frequent nouns from Medline. The second method implements an alignment-based technique to learn frequent generic patterns that indicate a hyponymy/hypernymy relationship between a pair of noun phrases. We then apply these patterns to Medline to collect frequent hypernyms as potential biomedical categories.

**Results:**

We study and compare these two alternative sets of terms to identify semantic categories in Medline. We find that both approaches produce reasonable terms as potential categories. We also find that there is a significant agreement between the two sets of terms. The overlap between the two methods improves our confidence regarding categories predicted by these independent methods.

**Conclusions:**

This study is an initial attempt to extract categories that are discussed in Medline. Rather than imposing external ontologies on Medline, our methods allow categories to emerge from the text.

## Background

Medline is a large and fast growing collection of biomedical documents containing over 22 million records as of January 2012. Finding meaningful categories of entities in such a large source of textual information is a useful task. These categories can be useful in constructing machine learning features, developing semantic representations for the text, finding smoothing or back-off probabilities for NLP tasks, and extracting information.

There are several humanly defined ontologies relevant to Medline. The Unified Medical Language System (UMLS^®^) Semantic Network is one such source. It consists of 134 semantic types and 54 semantic relationships between the types. Several users have modified UMLS to address their specific needs. One example is SemCat [1] which contains over 5 million entities and is based on subsets of UMLS enriched with additional categories from GENIA [2], UniProt [3], the Gene Ontology (GO) [4], Entrez Gene [5], and other knowledge sources. It is an attempt to define some important categories in the area of molecular biology.

The main goal of this study was to automatically identify semantic categories guided by the Medline text itself. We describe two methods for automatic acquisition of potential semantic categories from Medline. One approach is a simple statistical method that uses part-of-speech information, frequency information, and a set of well-defined multiword biomedical phrases to detect biomedical terms that are plausible categories in Medline. Another approach is a statistical learning method which identifies lexical patterns that describe hyponymy/hypernymy relationships between pairs of noun phrases (NP). This method detects a list of 40 such generic patterns for Medline. We then apply these patterns to all of Medline and collect the most frequent hypernyms. These hypernyms represent another source of potential semantic categories that emerge from Medline data. We compare these two sets of terms and find a significant overlap between them.

We now preview the contents of the paper. In the next section we describe our two methods. The first subsection concentrates on extracting categories using our statistical method. The second subsection describes a pattern-based approach for extracting categories. There we describe an alignment-based approach to generating patterns, and then we use these patterns to extract categories. In the Results section we compare categories extracted by the statistical and pattern-based methods. We also compare categories extracted by our methods with UMLS categories, and examine the patterns identified by the alignment-based approach. Finally we discuss our findings and draw conclusions.

## Methods

### A simple statistical method for category extraction

Here we describe a statistical method that is based on part-of-speech information and frequency counts. We first tagged the Medline text using the MedPost part of speech tagger [6]. We then normalized the nouns (lowercased and singularized) and extracted a list of nouns that satisfy the following three criteria:

• The token is tagged as a noun in more than 10,000 Medline documents;

• The token is a stand-alone noun in at least 1,000 Medline documents;

• The token is the headword of at least 10/50/100 distinct multiword biomedical noun phrases.

The condition of appearing in more than 10,000 Medline documents is designed to restrict attention to the most important categories. This condition was satisfied by 4,643 nouns.

By a stand-alone noun in condition two we mean a noun that is not part of a longer noun phrase, i.e. a noun that is not preceded or followed by another noun or adjective, with or without a determiner. The second condition is to ensure that the noun is meaningful on its own without any other qualifiers. One would expect this of a category name. This condition was satisfied by 13,613 nouns, among which 4,457 nouns also satisfied the first condition. We will denote this set of 4,457 nouns as *FN*. Many nouns ruled out by the second condition are part of a multiword concept, such as blot in 'western blot', spectrometry in 'mass spectrometry', escherichia in 'escherichia coli', or cytometry in 'flow cytometry'.

The third condition requires a set of well-defined multiword biomedical phrases. By multiword we mean more than one token not counting a determiner. For this purpose we use high-quality multiword UMLS phrases that are present in Medline and an additional set of multiword phrases automatically extracted from Medline. This additional set of multiword phrases extracted from Medline is designed to be of similar quality as UMLS phrases. Details on the construction of this set of phrases may be found in [7]. We have a total of 676,254 phrases in this set of well-defined multiword biomedical phrases, from which we extracted all headwords and their frequencies of occurrence. We denote the resulting set of headwords by *HN*.

A good category name with a rich content should be able to take multiple modifiers. This motivates condition three. We examined *HN *at 3 different cut-off points: headwords that appear in 10 or more, 50 or more, and 100 or more distinct phrases. We will refer to these sets as *HN10*, *HN50*, and *HN100*. The sets *HN10/HN50/HN100 *contain 7,658/1,447/666 head nouns respectively, among which 3,145/1,199/601 also satisfy the first two conditions. We denote these sets of nouns that satisfy all three conditions by *FHN10*, *FHN50*, and *FHN100*.

To summarize, condition 1 ensures that the noun is frequent, conditions 2 ensures that the noun is meaningful on its own and can be used without any other qualifiers, and condition 3 ensures that the noun is able to take multiple different modifiers.

### Pattern-based category extraction

#### Alignment-based pattern generation

Our second approach is a statistical learning method which identifies lexical patterns that describe a hyponymy/hypernymy relationship between a pair of noun phrases. We approximate the hyponymy/hypernymy relationship by the more general narrower/broader relationship defined in the UMLS Metathesaurus^® ^file "MRREL.RRF" (http://www.nlm.nih.gov/research/umls/).

We process the UMLS "MRREL.RRF" file to extract 179,285 pairs of terms that form the narrower/broader relationship and use them to extract sentences from Medline containing these pairs of terms. We denote the resulting set of 13,741,551 sentences by *S*.

In order to apply a statistical pattern generating method, we define features for each sentence. Given a sentence *s *∈ *S *that contains a pair of terms X/Y that have a narrower/broader relationship (or vice versa), we replace the narrower term with X, the broader term with Y, and represent the sentence as

(2.1) $$s=w_{1}\cdots w_{k}Xw_{l}\cdots w_{m}Yw_{n}\cdots w_{r}$$

where the *w_i_'s *represent all the tokens of *s *other than those contained in X and Y, and all the elements are in the order they appear in *s *. Then we extract a set of features associated with sentence *s *as follows.

• All ordered word pairs *w_i _w_j _*before term *X *(*i *<*j *≤ *k*).

• All ordered pairs *w_i _X *(*i *≤ *k*).

• All ordered pairs *Xw_i _*(*l *≤ *i *≤ *m*).

• All ordered word pairs *w_i_w_j _*between *X *and *Y *(*l *≤ *i *≤ *j *≤ *m*).

• All ordered pairs *w_i_Y *(*l *≤ *i *≤ *m*).

• All ordered pairs *Yw_i _*(*n *≤ *i*).

• All ordered word pairs *w_i _w_j _*after term *Y *(*n *≤ *i *<*j*).

• A pair *(NX *, *BY *) if *X *is a narrower concept or *(BX *, *NY *) if *X *is a broader concept.

We represent each sentence *s *as a vector *v_s _*with the features defined above and denote the set of all such vectors as *V *. We randomly select a vector ψ∈*V *and use it as a query vector over the set *V *. Using a cosine similarity score we rank vectors from the highest to the lowest. We then consider only the top *N *ranking vectors, where the number *N *is defined as follows

(2.2) $$\begin{array}{c} N=\underset{50\leq i<300}{\text{arg}\text{max}}{\mathrm{\Delta i}}_{},\\ \text{where}\;\Delta_{i}=Score\left(i\right)-Score\left(i+1\right) \end{array}$$

If Δ*_N _*<0.01 a different query vector is selected. The drop off value of 0.01 was chosen empirically.

We denote these top *N *retrieved vectors as

(2.3) $$R={\left\{r_{i}\right\}}_{i=1}^{N}$$

We then chose the 5 sentences $\left\{r_{\frac{5N}{10}},r_{\frac{6N}{10}},r_{\frac{7N}{10}},r_{\frac{8N}{10}},r_{\frac{9N}{10}}\right\}$ from the bottom half of (2.3).

We did not choose from the top sentences because they tended to be nearly identical, obscuring any generalizable pattern.

The boundaries for *N *are set for the following reasons. We need *N *to be at least 50 because we choose 5 sentences spread over the bottom half of set *R *starting from the median ranked sentence. On the other hand, if there is no considerable score drop off observed within the first 300 top ranking sentences, then sentences tend to be nearly identical.

We further process these 5 sentences by considering only the word order features with relative frequencies above 0.8 in set *R*. Other features are dropped. For each of these sentences we start with form (2.1) and remove any token that does not appear in the selected features. This produces a reduced representation of the sentence. We then apply the multiple sequence alignment algorithm Clustal [8] to the reduced representations of the 5 sentences and collect the resulting pattern.

This procedure was carried out 5,000 times. This generated 850 unique patterns and on review we selected 40 patterns as the most useful for identifying the 'is a' relation.

These 40 patterns are presented in Table 1.

**Table 1:** List of 40 patterns generated by alignment-based method.

X is a Y	X is a potent Y
X are Y	X is the most common Y
X and other Y	X are rare Y
X as a Y	X is a widely used Y
X such as Y	X is an uncommon Y
X is an Y	X is an autosomal dominant Y
X as an Y	X is a form of Y
X is an important Y	X is one of the major Y
X a new Y	X is a chronic Y
X are the most common Y	X and other forms of Y
X is a rare Y	X is a broad spectrum Y
X is a novel Y	X is the primary Y
X is a major Y	X is a rare autosomal recessive Y
X is an essential Y	X is the most common type of Y
X was the only Y	X is the second most common Y
X was the most common Y	X are the most frequent Y
X is a common Y	X is the most widely used Y
X is a new Y	X is the most frequent Y
X is a complex Y	X is the most common primary Y
X is an effective Y	X is one of the major Y

We illustrate this technique with a worked out example. A sentence

*Quinoproteins are a big class of oxyreductive agents occurring in **bacteria **and other **organisms***.

is randomly selected as a query vector. The terms *bacteria *and *organisms *represent a pair describing a narrower/broader relationship from UMLS and are replaced by X and Y. Sentences in *V *are ranked using the cosine similarity score. The 5 sentences below are chosen for multiple sequence alignment:

*Spouse abuse and other domestic violence*.

*Effects of anticoagulants and other drugs*.

[Epidemiology of rabies virus and other lyssaviruses]

*Biosynthesis of Cholesterol and Other Sterols*.

*Alcohol and other drug dependencies*.

Table 2 demonstrates the representation of these sentences in terms of X and Y. Features that occurred with a relative frequency above 0.8 over the set R are the following ordered pairs:

**Table 2:** Sentences containing pairs of terms with narrower/broader relationship, with narrower term replaced by X and wider term replaced by Y.

	Pattern			
		
	X	and other	Y	
	**Spouse abuse**	**and other**	**domestic violence**	

Effects of	anticoagulants	and other	drugs	
Epidemiology of	rabies virus	and other	lyssaviruses	
Biosynthesis of	Cholesterol	and other	Sterols	
	Alcohol	and other	drug	dependencies

*X and; X other; and other; and Y; other Y*.

Other features are dropped. The multiple sequence alignment algorithm applied to these 5 sentences detects the 'X and other Y' as a pattern.

#### Using patterns to extract categories

We now utilize these 40 generic patterns that are useful for identifying the 'is a' relationship between a pair of NPs to detect potential semantic categories in Medline. For example, consider the sentence:

*The unique action of propranolol and other beta blockers in lowering raised arterial pressure is discussed*.

matches the pattern 'X and other Y'. In this example, beta blockers can be identified as a potential category and propranolol as an instance of the beta blockers category.

For each of these patterns 'X string Y', we extract Medline sentences containing them such that X and Y are noun phrases. For all strings except 'such as', the noun phrase beginning the pattern X is a hyponym of the noun phrase Y at the end of the pattern. For generality, we will refer to noun phrases at position X as an instance of a category described by noun phrase at position Y.

From the resulting set of 2.88 million sentences, we extracted pairs of noun phrases at positions X and Y and grouped the Y noun phrases by headwords. These headwords are potential semantic categories. Thus we acquired a set of 2,475 nouns, potential semantic categories that appeared in Medline sentences matching 'X string Y' patterns at least 50 times. We will refer to the set of 2,475 nouns as *PN_50_*.

The idea of using patterns is not new. It has been extensively studied in the literature [9,10], but mostly for populating a category, i.e. finding instances for a given category. Our observation is that one can use the patterns to detect both categories and instances.

## Results

### Comparison of categories detected by pattern-based and statistical methods

Using our statistical method we extracted sets of frequent biologically meaningful nouns *FHN*_100 _⊂ *FHN *_50 _⊂ *FHN*_10 _containing 601/1,199/3,145 tokens. Using our pattern-based method we extracted a set *PN_50 _*of 2, 475 nouns.

Here we compare the contents of sets extracted by the two different methods. We examine how many elements of sets *FHN10*, *FHN50*, and *FHN100 *are present in set *PN_50_*. Results are presented in Table 3. The first column of Table 3 describes how many elements are at the intersection of two sets, while the second column presents the percentage of sets *FHN10*, *FHN50*, and *FHN100 *found in *PN_50_*. We observe that 87% of the nouns predicted by our statistical method in *FHN100 *are also predicted by the pattern-based method. We also observe that 76% of nouns in *FHN50 *and 50% of nouns in *FHN10 *are present in *PN_50_*. Such highly significant agreement between these sets of nouns generated by two independent methods suggests a strong relationship between them and that both of them are valuable. Moreover, we can have increased confidence in those categories predicted by both methods.

**Table 3:** Counts, Percentage, and Significance of overlap between *FHN*_100 _⊂ *FHN*_50 _⊂ *FHN*_10 _and *PN*_50 _lists. Percentage is computed relative to *FHN *sets.

Set (size)	*PN_50 _(2,475)*		
	
	Counts	Percentage	-*Log*_10 _of *p*-value
*FHN_10 _(3,145)*	1,564	50%	2215
*FHN_50 _(1,199)*	915	76%	1483
*FHN_100 _(601)*	522	87%	885

### Comparison with UMLS Metathesaurus "MRREL.RRF" file

We described in the Methods section that we extracted 179,285 pairs of terms that form the narrower/broader relationship from UMLS "MRREL.RRF" file. The headwords of the broader noun phrases comprise 13,159 unique nouns. Not all of the original UMLS pairs have the 'is a' kind of relationship. We examined the set of Medline sentences containing the 40 'X string Y' patterns and found that 10,960 of the 13,159 headwords from UMLS pairs appeared as headwords of Y noun phrases in these sentences. Of these 10,960 headwords, 1,313 appear as headwords of Y noun phrases in Medline sentences matching 'X string Y' pattern 50 or more times. For comparison, using our pattern-based category extraction method we have found 2,475 headwords that appear more than 50 times in Medline sentences. Thus, we have nearly doubled the number of headwords that are identified as potential categories as compared to UMLS.

### Examining the patterns

Our pattern-based category extraction method generated a list of 2,475 nouns as potential semantic categories. Each of these nouns appeared in at least 50 Medline sentences matching the 'X string Y' ranging over the list of 40 patterns presented in Table 1. Now that we have the list of nouns in *PN_50 _*we observe that some patterns were more useful in generating these nouns than others. By counting how many nouns each of the patterns contributed to the final list of 2,475 nouns, we detect that the first 25 patterns are sufficient to acquire all nouns in the list. Moreover, the first 16 patterns are sufficient to generate 99% of the nouns in *PN_50_*, and top 7 patterns are sufficient to generate 96% of the nouns. These 7 patterns are: X is a Y, X are Y, X and other Y, X as a Y, X such as Y, X is an Y, X as an Y.

## Discussion

A useful by-product of category identification is a large amount of additional information available about each category. In addition to the category we obtain subcategories, instances of the category, and groupings of instances into subcategories. On the highest level we acquired a list of headwords of noun phrases, which are potential category names. The phrases with the same headword are subtypes of the category. For example, infection is identified as a potential category name. Some of the noun phrases that share the infection headword are:

*viral infections, fungal infections, bacterial infections, parasitic infections, chronic infections, genital infections, respiratory infections, pulmonary infections, streptococcal infections*.

These are types of infections. The X noun phrases are instances of category defined by the headword of Y and subcategory Y. Sample instances of the infection category are:

*hiv; mucormycosis; tuberculosis; actinomycosis; toxoplasmosis; coccidioidomycosis; histoplasmosis; pneumonia; lyme disease; influenza; malaria; aids; cryptococcosis; nocardiosis; onychomycosis; aspergillosis; syphilis*.

These instances can be further grouped by subcategory. For example:

varicella, herpes, zoster, rabies, measles, cytomegalovirus, aids, mumps, hcv, cmv, dengue infection

are detected as viral infections.

We developed and applied our methods on biomedical literature. However these methods are not constrained to biomedical text, and can be applied to other subject areas and corpora with two minor modifications. The statistical method requires a set of well-defined multiword biomedical phrases in Condition 3 to ensure that a noun can act as a headword of multiple different phrases. Such a list of well-defined meaningful phrases may not be available in other subject areas. In that case, one could simply consider noun phrases in a given corpora and extract all headwords and their frequencies of occurrence with different noun phrases. Naturally, the frequency threshold needs to be adjusted based on the corpus used. Another knowledge source that is corpus specific is the set of UMLS pairs we used to generate patterns. Such source may not be available for other corpora. However, there is no need to generate the patterns again. Most of the patterns that we generated are quite general and can be used for different corpora. Very few patterns, such as 'X is an autosomal dominant Y' or 'X is a rare autosomal recessive Y' are subject specific. Moreover, as our analysis indicates the top 25 patterns acquire 99% of the nouns in *PN*_50_. Thus one could use these 25 general patterns and apply them to a different domain. These patterns are generic and can be used in any subject area, regardless of the fact that biomedical data was used to generate them.

## Conclusions and future work

Given a large source of textual information we studied how to automatically find meaningful categories of entities in such a source. We examined two methods for finding the categories. One is a simple statistical method that extracts frequent headwords of noun phrases from Medline. The second is an alignment-based method that generates patterns for detecting hyponymy/hypernymy relationships and uses these patterns to extract frequent hypernyms from Medline. We compared the results of these two methods and found a significant overlap between them.

These two methods are based on different aspects and characteristics of the data, therefore we do not expect a similar bias to be introduced into their analyses. Considering the overlap between the two methods improves our confidence regarding the categories predicted by both methods independently, compared to categories predicted only by one method and not the other.

This study is an initial attempt to automatically extract the categories that are discussed in Medline. In the future we plan to further analyze these categories. We plan to use machine learning to try to distinguish the terms that are in the intersection of the two methods from the terms that are not. In this approach one may use the criteria for identifying potential categories with the statistical and pattern-based methods as features. In addition, there are potentially useful properties, such as whether the term is content-bearing as defined in [11] and the frequency of the term in PubMed queries.

## Competing interests

The authors declare that they have no competing interests.

## Authors' contributions

WJW suggested the study and all authors equally participated in the design of the study. LY and WK implemented the methods. LY and DCC drafted the manuscript. All authors read and approved the final manuscript.
